# Clinical Characteristics and Electrophysiological Biomarkers of Parkinson's Disease Developed From Essential Tremor

**DOI:** 10.3389/fneur.2020.582471

**Published:** 2020-10-29

**Authors:** Xuemei Wang, Zhentang Cao, Genliang Liu, Zhu Liu, Ying Jiang, Huizi Ma, Zhan Wang, Yaqin Yang, Huimin Chen, Tao Feng

**Affiliations:** ^1^Center for Movement Disorders Disease, Department of Neurology, Beijing Tiantan Hospital, Capital Medical University, Beijing, China; ^2^China National Clinical Research Center for Neurological Diseases, Beijing, China; ^3^Parkinson's Disease Center, Beijing Institute for Brain Disorders, Beijing, China

**Keywords:** Parkinson's disease, essential tremor, tremor, clinical, electrophysiological

## Abstract

**Background and Objective:** Parkinson's disease developed from essential tremor (ET-PD) is a distinct clinical syndrome that is different from essential tremor (ET) and Parkinson's disease (PD). There is currently a lack of research on ET-PD. Tremor characteristics (amplitude and frequency) are primary quantitative indexes for diagnosing and monitoring of tremors. In this study, we aimed to explore specific clinical and electrophysiological biomarkers for the identification of ET-PD.

**Methods:** The study included patients with ET-PD (*n* = 22), ET (*n* = 42), and tremor-dominant PD (t-PD, *n* = 47). We collected demographic data, clinical characteristics (including motor and non-motor symptoms), and tremor analysis. The frequency, amplitude, contracting patterns of resting tremor and postural tremor were collected. The analysis of ET-PD and ET/t-PD was compared. The receiver operating characteristic (ROC) curve was used to analyze the electrophysiological features in distinguishing ET-PD from ET or t-PD.

**Results:** Compared with ET, hyposmia, bradykinesia, rigidity, postural abnormality, and resting tremor were more common in the ET-PD group (*P* = 0.01, 0.003, 0.001, 0.001, 0.019, respectively). The postural tremor frequencies of the head, upper limbs, and lower limbs were significantly lower in the ET-PD than in the ET (*P* = 0.007, 0.003, 0.035, respectively), which were the most appropriate variables for distinguishing ET-PD from ET (AUC: 0.775, 0.727, and 0.701, respectively). Compared with t-PD, bradykinesia, rigidity, postural abnormality (both *P* < 0.001), and resting tremor (*P* = 0.024) were less common in the ET-PD. The postural tremor amplitudes of the head and upper limbs were significantly higher in the ET-PD than in the t-PD (*P* = 0.022, 0.001, respectively), which were the most appropriate variables for distinguishing ET-PD from t-PD (AUC: 0.793 and 0.716).

**Conclusions:** Hyposmia and electrophysiological biomarkers (postural tremor frequencies and amplitudes) help early recognition of ET-PD.

## Introduction

Essential tremor (ET) and idiopathic Parkinson's disease (PD) are two of the most common movement disorders. PD's motor and non-motor features may overlap with ET, making it difficult to distinguish them based on clinical characteristics (1). For example, besides the typical resting tremor, patients with PD also often exhibit postural tremor, which is more often observed in patients with ET (2). In turn, in a population-based setting, resting tremor is a common clinical feature in patients with ET, and the prevalence can reach nearly 50% (3). ET patients have significant movement slowness compared to healthy controls, and a considerable number of ET patients have movement abnormalities similar to those observed in PD patients (4). Depression, anxiety, cognitive disorders, and family history of tremors/PD were similar in both patient groups (5, 6). Thus, clinical and experimental evidence indicates that there are similarities between ET and PD.

It has been reported that patients with ET seem to be about four to five times more likely to develop PD than the general population (7). ET that eventually develops into PD is called essential tremor–Parkinson's disease (ET-PD) (8). Clinically, patients with ET-PD can exhibit different types of tremor, including PD-associated resting tremor and ET-associated postural tremor. Whether ET-PD is a co-occurrence of two relatively common pathologies or if ET is a prodromal stage of PD in some patients is yet to be elucidated. Electromyographic (EMG) examination is a useful tool for tremor analysis and can characterize different tremor types according to tremor amplitude, frequency, and pattern (9). Interestingly, a recent study demonstrated that patients with ET-PD, unlike patients with tremor-dominant PD (t-PD), exhibited the synchronous pattern of resting tremor (10). However, the exact phenomenology and etiology of this finding remain unclear. Besides, due to the scarce clinical data for ET-PD, it remains unknown which clinical and tremor features can differ it from ET or t-PD. This also means that the objective factors to predict the progression of ET to PD are limited. Thus, it is difficult to obtain an accurate diagnosis of ET-PD in the early stages. Considering that electrophysiological methods can effectively characterize tremor, we systematically analyzed differences in clinical and neurophysiological features between patients with ET-PD and ET/t-PD to determine clinical and electrophysiological markers of ET-PD in order to obtain useful and targeted treatment in the early stage of ET-PD.

## Materials and Methods

### Patients

From March 2018 to October 2019, patients with ET-PD (*n* = 22), ET (*n* = 42), and t-PD (*n* = 47) were recruited from Beijing Tiantan Hospital. A diagnosis of PD was made according to the Movement Disorders Society Clinical Diagnostic Criteria for Parkinson's disease in 2015 (11). The t-PD group's inclusion criteria were as follows: patients had at least one-limb resting or postural tremor, and the age of onset was >50 years old. Exclusion criteria for the t-PD group were as follows: (1) secondary parkinsonism; (2) atypical PD; and (3) severe heart disease, liver or kidney disease, or any other chronic disease meaning that the patient could not complete the examinations. A diagnosis of ET was made using criteria from the Movement Disorder Society Tremor Investigation Group (12). A diagnosis of ET was not assigned if bradykinesia, rigidity, or resting tremor appeared within 5 years of the onset of tremor attributed to ET.

Inclusion criteria of the ET-PD (13) group were as follows: (1) ET had been diagnosed at least 5 years before the PD diagnosis and (2) when they had received a previous diagnosis of ET, patients exhibited significant characteristics of postural tremor without any symptoms or signs of PD. Exclusion criteria for ET-PD were as follows: (1) age at PD diagnosis <40 years old; (2) atypical PD or secondary parkinsonism; and (3) patients with a history of postural or action tremor <5 years.

For all patients, we collected demographic data (sex and age), clinical data including motor symptoms, non-motor symptoms, age of onset of PD, disease duration, tremor location, tremor pattern, past medical history, family history, medication history, and anti-tremor drug responsiveness. The Unified Parkinson's Disease Rating Scale (UPDRS) and the Hoehn & Yahr (H&Y) scale in an “off” phase (>12 h after the last dose of dopaminergic medication; >24 h after anticholinergics or β-blockers) were evaluated for the t-PD and ET-PD groups (14).

This study was approved by the Ethics Committee of Beijing Tiantan Hospital and was performed in accordance with the Declaration of Helsinki. All patients provided informed consent.

### Definition of Non-motor Features

Constipation was defined according to the Rome III diagnostic criteria (15).

Olfactory function was evaluated with the 12-item Sniffin' Sticks test (Burghart Messtechnik GmbH, Wedel, Germany). Patients were considered to have rapid eye movement sleep behavior disorder (RBD) when they fulfilled the criteria determined by the REM Sleep Behavior Disorder Screening Questionnaire (RBDSQ) (16).

Depression was assessed using the Hamilton Depression Rating Scale (HAMD) (17), and anxiety was assessed using the Hamilton Anxiety Rating Scale (HAMA) (18).

Cognitive status was assessed using the Mini-Mental State Examination (MMSE) scale (19) and the Montreal Cognitive Assessment (MoCA) scale (20).

### Tremor Analysis

Tremor was recorded using an electromyography evoked potential meter (Nicolet EDX, USA), which has four pairs of surface electrodes and two piezoresistive accelerators. The recording electrodes were placed on the muscle bellies of the flexor carpi and extensor carpi of both forearms and lower limbs. The reference electrode was placed on the corresponding tendon, and the accelerator was fixed at the proximal end of the third metacarpal of the ipsilateral hand. For head tremor examination, the recording electrode was placed at the midpoint of the sternocleidomastoid muscle, and the reference electrode was placed in the supraclavicular fossa. The EMG parameters were as follows: amplifier sensitivity at 100 μV/div, sweep speed at 100 ms/div, and a filter width of 10.0 Hz−10.0 kHz; piezoresistive accelerometer sensitivity at 2.2 mV/g and a filter width in the range 0.5–30.0 Hz.

Tremor was assessed under the following six conditions: (1) resting tremor of the head was assessed while patients sat in an armchair, leaning their head and back against the chair back, and relaxing their head; (2) resting tremor in the upper limbs was assessed while patients sat in an armchair with the forearms and hands completely rested on the armrests; (3) resting tremor in the lower limbs was assessed while patients sat in an armchair, with their feet placed flatly on the ground, and while they were utterly relaxed; (4) postural tremor in the head was assessed while patients sat in an armchair, keeping their head upright; (5) postural tremor in the upper limbs was assessed while patients sat in an armchair with wrists/fingers outstretched on a horizontal plane; and (6) postural tremor in the lower limbs was assessed while patients sat in an armchair with both sides of their toes touching the ground and with their heels hanging. Each measurement session lasted for 30 s. The EMG data were analyzed using the TRAS system (21). All tests were performed during the patients' “off” state (22). Resting and postural tremor frequency, tremor amplitude, and systolic patterns were recorded.

### Statistical Analysis

Analyses were performed using SPSS 24.0 software (SPSS Inc., Chicago, IL, USA). Graphs were delineated by using Prism 7.0 (GraphPad software, La Jolla, CA, USA). The normality of distribution of continuous variables was tested by one-sample Kolmogorov–Smirnov test. Continuous variables with normal distribution were presented as mean ± standard deviation (SD). The differences in continuous variables between the ET-PD and ET/t-PD groups were assessed using independent samples Student's test when the data were normally distributed and using Mann–Whitney *U* test if the data were not normally distributed. A chi-square test was used to compare categorical variables between the groups. Sensitivity and specificity for differentiating ET-PD from ET or t-PD were calculated using the optimal cutoff value determined by receiver operating characteristic (ROC) curve analysis. The optimum cutoff value for the ROC curve was determined using the Youden Index. Differences with a *P* < 0.05 were considered to be statistically significant.

## Results

### Comparison of Demographic, Clinical, and Electrophysiological Features Between Patients With Essential Tremor–Parkinson's Disease and Essential Tremor

#### Differences in Demographic Variables

The demographic data are shown in Table 1. Compared with the ET group, there was no difference in the ET-PD group regardless of sex or age.

**Table 1:** Comparison of demographic, clinical, and electrophysiological features between patients with ET-PD and ET.

**Variables**	**ET-PD (*n* = 22)**	**ET (*n* = 42)**	***P*-value**
**Demographic characteristics**			
Age, years (mean ± SD)	64.14 ± 9.26	59.10 ± 10.47	0.56
Sex: No. men/women	10/12	23/19	0.48
**Clinical characteristics**			
Family history (Postural/kinetic tremor), *n* (%)	13 (59)	27 (63)	0.683
Age at onset of ET, years (mean ± SD)	50.86 ± 12.11	41.83 ± 16.62	0.025^*^
Disease duration of ET, years (mean ± SD)	13.27 ± 9.99	17.40 ± 11.47	0.054
Disease duration from ET to PD onset, years (mean ± SD)	12.30 ± 2.18	-	-
Constipation, *n* (%)	7 (32)	9 (21)	0.362
Hyposmia, *n* (%)	6 (27)	2 (5)	0.01^*^
RBD, *n* (%)	8 (36)	7 (17)	0.077
HAMD (mean ± SD)	12.88 ± 6.90	9.50 ± 7.43	0.122
HAMA (mean ± SD)	13.88 ± 4.39	9.40 ± 8.03	0.114
MMSE (mean ± SD)	23.38 ± 7.96	22.80 ± 8.77	0.572
MoCA (mean ± SD)	17.50 ± 7.71	18.70 ± 8.06	0.909
Bradykinesia, *n* (%)	10 (45.50)	5 (12)	0.003^*^
Rigidity, *n* (%)	7 (32)	1 (2)	0.001^*^
Postural abnormality, *n* (%)	7 (32)	1 (2)	0.001^*^
Drinking responsiveness, *n* (%)	5 (23)	18 (43)	0.111
Arotinolol responsiveness, *n* (%)	6 (37.50)	25 (60)	0.014^*^
Unilateral disease onset, *n* (%)	13 (59)	13 (31)	0.029^*^
Bilateral disease onset, *n* (%)	9 (41)	29 (69)	0.029^*^
Upper limb tremor, *n* (%)	22 (100)	42 (100)	-
Lower limb tremor, *n* (%)	15 (68)	9 (21)	<0.001^*^
Head tremor, *n* (%)	10 (45.50)	18 (43)	0.842
Mandibular tremor, *n* (%)	5 (23)	2 (5)	0.029^*^
Resting tremor, *n* (%)	16 (73)	20 (48)	0.019^*^
Postural tremor, *n* (%)	22 (100)	41 (98)	0.466
**Electrophysiological description**			
***Resting tremor***			
Frequency (Hz)			
Head	4.78 ± 0.25	5.92 ± 0.98	0.058
Upper limbs	4.56 ± 0.87	5.33 ± 1.20	0.064
Lower limbs	4.72 ± 0.88	-	-
Amplitude (μV)			
Head	352.00 ± 98.57	309.10 ± 105.55	0.525
Upper limbs	1104.27 ± 435.39	689.11 ± 313.05	0.132
Lower limbs	469.23 ± 313.38	-	-
Synchronous patterns, *n* (%)			
Head	4/5 (80)	5/5 (100)	0.292
Upper limbs	5/16 (31)	9/18 (50)	0.332
Lower limbs	5/10 (50)	0	-
Alternating patterns, *n* (%)			
Head	0/5 (0)	0/5 (0)	-
Upper limbs	8/16 (50)	8/18 (44)	0.877
Lower limbs	3/10 (30)	0	-
Synchronous and alternating patterns, *n* (%)			
Head	1/5 (20)	0/5 (0)	0.292
Upper limbs	3/16 (19)	1/18 (6)	0.212
Lower limbs	2/10 (20)	0	-
***Postural tremor***			
Frequency (Hz)			
Head	4.50 ± 0.45	5.41 ± 1.01	0.007^*^
Upper limbs	5.15 ± 1.04	6.13 ± 1.49	0.003^*^
Lower limbs	5.10 ± 1.37	6.35 ± 0.91	0.035^*^
Amplitude(μV)			
Head	474.07 ± 166.99	661.97 ± 242.99	0.738
Upper limbs	1,419.70 ± 426.17	922.91 ± 135.29	0.364
Lower limbs	1,039.23 ± 221.46	504.96 ± 263.43	0.132
Synchronous patterns, *n* (%)			
Head	5/7 (71)	14/16 (87.50)	0.499
Upper limbs	4/22 (18)	22/40 (55)	0.005^*^
Lower limbs	8/11 (73)	3/3 (100)	0.308
Alternating patterns, *n* (%)			
Head	0/7 (0)	1/16 (6.25)	0.349
Upper limbs	10/22 (46)	9/40 (22.50)	0.061
Lower limbs	1/11 (9)	0/3 (0)	0.588
Synchronous and alternating patterns, *n* (%)			
Head	2/7 (29)	1/16 (6.25)	0.144
Upper limbs	8/22 (36)	9/40 (22.50)	0.242
Lower limbs	2/11 (18)	0/3 (0)	0.425

ET-PD, Parkinson's disease developed from essential tremor; ET, essential tremor; RBD, rapid eye movement sleep behavioral disorder; HAMD, Hamilton Depression Scale; HAMA, Hamilton Anxiety Rating Scale; MMSE, Mini-Mental State Examination; MoCA, Montreal Cognitive Assessment; UPDRS, Unified Parkinson's Disease Rating Scale.

* *This P-value indicates a statistically significant difference*.

#### Differences in Clinical Characteristics

All clinical data are shown in Table 1. The ET-PD group had an older mean age at onset of ET than the ET group (*P* = 0.025). The average latency of ET to PD diagnosis in the ET-PD group was 12.30 ± 2.18 years. Hyposmia was significantly more common in the ET-PD group than the ET group (*P* = 0.01), but no other significant differences in non-motor features were found between these two groups. Concerning motor features, the proportion of patients with ET-PD with asymmetric motor symptoms (59%) was higher than that in the ET group (*P* = 0.029). Bradykinesia, rigidity, and postural abnormalities were more common in the ET-PD group than the ET group (*P* = 0.003, 0.001, 0.001, respectively). Resting tremor was found in 73% of the ET-PD group, which was a significantly higher proportion than that in the ET group (*P* = 0.019). Lower limb tremor was significantly more common in the ET-PD group than that in the ET group (*P* < 0.001), as was mandibular tremor (*P* = 0.029).

#### Difference in Electrophysiological Results

**Figure 1:**
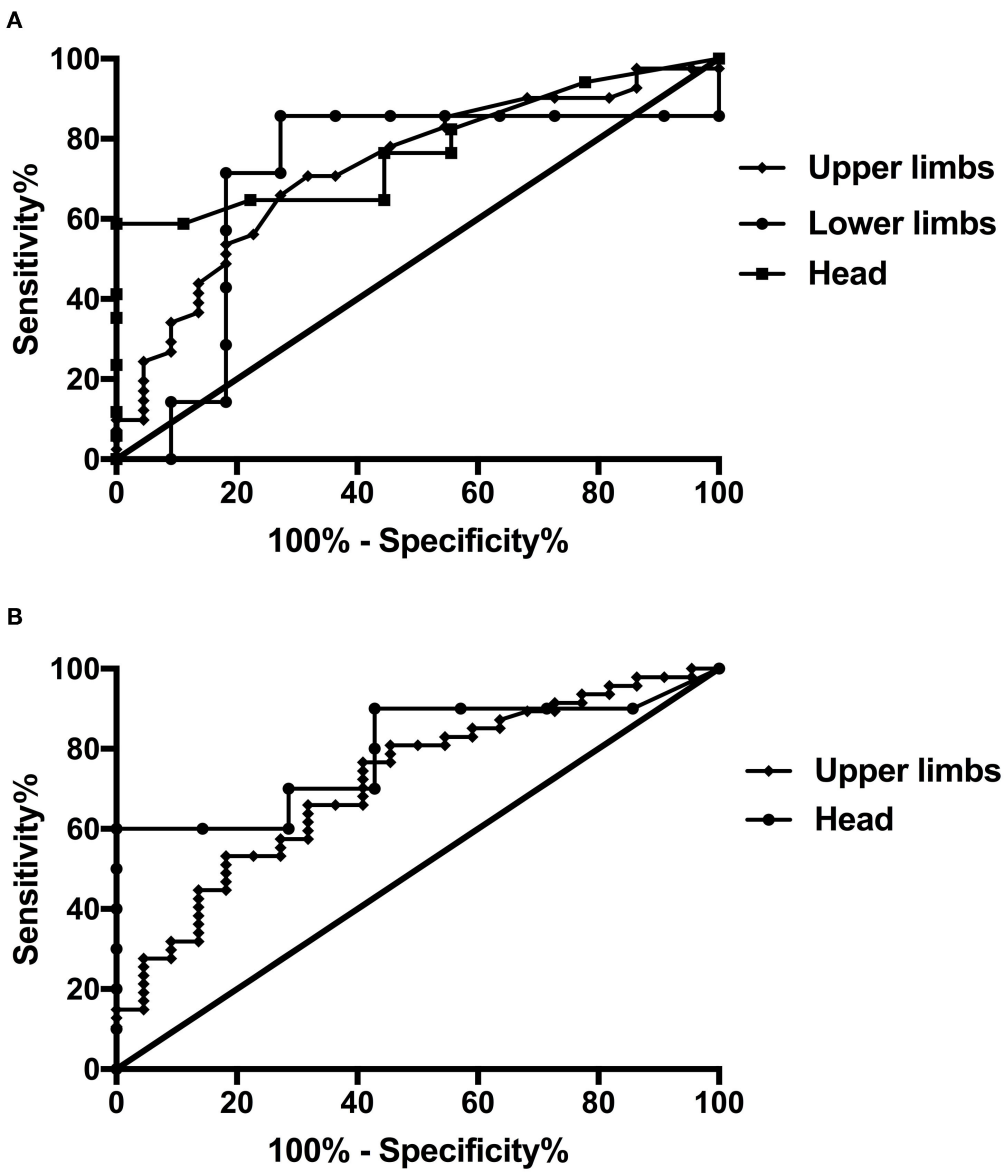
Receiver operating characteristic (ROC) curve differentiating essential tremor–Parkinson's disease (ET-PD) from essential tremor (ET) or tremor-dominant Parkinson's disease (t-PD). **(A)** ROC curve of postural tremor frequency to differentiate ET-PD from ET. **(B)** ROC curve of postural tremor amplitude to differentiate ET-PD from t-PD.

Postural tremor was observed in almost all patients in the ET-PD and ET groups. However, the postural tremor frequency differed between the ET-PD and ET groups. The postural tremor frequencies of the head, upper limbs, and lower limbs were significantly lower in the ET-PD group than those in the ET group (*P* = 0.007, 0.003, 0.035, respectively; Table 1). The cutoff value of head postural tremor frequency to distinguish patients with ET-PD from ET was 5.20 Hz, with a sensitivity of 67% and specificity of 100%. The cutoff value of postural tremor frequency to distinguish patients with ET-PD from ET was 5.45 Hz for the upper limbs and 5.98 Hz for the lower limbs, with a sensitivity of 100% and specificity of 83%, respectively (**Table 3**, Figure 1A). Furthermore, upper limb postural tremor patterns were less synchronous in the ET-PD group than those in the ET group (Table 1).

### Comparison of Demographic, Clinical, and Electrophysiological Features Between Patients With Essential Tremor–Parkinson's Disease and Tremor-Dominant Parkinson's Disease

#### Differences in Demographic Variables

The demographic data are shown in Table 2. Compared with the t-PD group, there was no difference in the ET-PD group regardless of sex or age.

**Table 2:** Comparison of demographic, clinical, and electrophysiological features between patients with ET-PD and t-PD.

**Variables**	**ET-PD (*n* = 22)**	**t-PD (*n* = 47)**	***P*-value**
**Demographic characteristics**			
Age, years (mean ± SD)	64.14 ± 9.26	63.28 ± 7.01	0.07
Sex: No. men/women	10/12	24/23	0.67
**Clinical characteristics**			
Family history (Postural/kinetic tremor), *n* (%)	13 (59)	9 (19)	0.001^*^
Age at onset of PD, years (mean ± SD)	63.16 ± 9.62	56.51 ± 8.05	0.004^*^
Disease duration, years (mean ± SD)	13.27 ± 9.99	6.83 ± 4.53	0.008^*^
Constipation, *n* (%)	7 (32)	39 (83)	<0.001^*^
Hyposmia, *n* (%)	6 (27)	25 (53)	0.044^*^
RBD, *n* (%)	8 (36)	30 (64)	0.033^*^
HAMD (mean ± SD)	12.88 ± 6.90	11.13 ± 9.02	0.774
HAMA (mean ± SD)	13.88 ± 4.39	12.84 ± 7.34	0.920
MMSE (mean ± SD)	23.38 ± 7.96	26.00 ± 3.09	0.777
MoCA (mean ± SD)	17.50 ± 7.71	22.19 ± 4.71	0.168
Bradykinesia, *n* (%)	10 (45.50)	47 (100)	<0.001^*^
Rigidity, *n* (%)	7 (32)	43 (91.5)	<0.001^*^
Postural abnormality, *n* (%)	7 (32)	45 (96)	<0.001^*^
UPDRS part 3 (mean ± SD)	31.00 ± 1.41	38.39 ± 17.71	0.661
Hoehn–Yahr (mean ± SD)	2.41 ± 0.42	2.75 ± 0.85	0.054
Dopaminergic responsiveness, *n* (%)	7 (32)	30 (64)	0.013^*^
Unilateral disease onset, *n* (%)	13 (59)	47 (100)	<0.001^*^
Bilateral disease onset, *n* (%)	9 (41)	0 (0)	<0.001^*^
Upper limb tremor, *n* (%)	22 (100)	44 (94)	0.226
Lower limb tremor, *n* (%)	15 (68)	27 (57)	0.593
Head tremor, *n* (%)	10 (45.50)	3 (6)	<0.001^*^
Mandibular tremor, *n* (%)	5 (23)	22 (47)	0.038^*^
Resting tremor, *n* (%)	16 (73)	42 (89)	0.024^*^
Postural tremor, *n* (%)	22 (100)	47 (100)	-
**Electrophysiological description**			
***Resting tremor***			
Frequency (Hz)			
Head	4.78 ± 0.25	4.63 ± 0.56	0.887
Upper limbs	4.56 ± 0.87	4.47 ± 0.60	0.947
Lower limbs	4.72 ± 0.88	4.21 ± 0.55	0.336
Amplitude (μV)			
Head	352.00 ± 98.57	318.56 ± 184.14	0.646
Upper limbs	1,104.27 ± 435.39	703.53 ± 112.51	0.464
Lower limbs	469.23 ± 313.38	426.22 ± 74.97	0.776
Synchronous patterns, *n* (%)			
Head	4/5 (80)	5/9 (56)	0.360
Upper limbs	5/16 (31)	6/37 (16)	0.215
Lower limbs	5/10 (50)	10 (48)	0.595
Alternating patterns, *n* (%)			
Head	0/5 (0)	1/9 (11)	0.439
Upper limbs	8/16 (50)	23/37 (62)	0.409
Lower limbs	3/10 (30)	7 (33)	0.560
Synchronous and alternating patterns, *n* (%)			
Head	1/5 (20)	3/9 (33)	0.597
Upper limbs	3/16 (19)	8/37 (22)	0.813
Lower limbs	2/10 (20)	4 (19)	0.950
**Postural tremor**			
Frequency (Hz)			
Head	4.50 ± 0.45	4.40 ± 0.79	0.850
Upper limbs	5.15 ± 1.04	4.96 ± 0.70	0.948
Lower limbs	5.10 ± 1.37	4.84 ± 0.79	0.924
Amplitude(μV)			
Head	474.07 ± 166.99	293.35 ± 174.01	0.022^*^
Upper limbs	1,419.70 ± 426.17	787.48 ± 197.85	0.001^*^
Lower limbs	1,039.23 ± 221.46	740.85 ± 126.07	0.157
Synchronous patterns, *n* (%)			
Head	5/7 (71)	10/11 (91)	0.280
Upper limbs	4/22 (18)	16/42 (38)	0.103
Lower limbs	8/11 (73)	18/31 (58)	0.390
Alternating patterns, *n* (%)			
Head	0/7 (0)	0/11 (0)	-
Upper limbs	10/22 (46)	12/42 (29)	0.177
Lower limbs	1/11 (9)	5/31 (16)	0.567
Synchronous and alternating patterns, *n* (%)			
Head	2/7 (29)	1/11 (9)	0.280
Upper limbs	8/22 (36)	14/42 (33)	0.808
Lower limbs	2/11 (18)	8/31 (26)	0.610

ET-PD, Parkinson's disease developed from essential tremor; t-PD, tremor-dominant Parkinson's disease; RBD, rapid eye movement sleep behavioral disorder; HAMD, Hamilton Depression Scale; HAMA, Hamilton Anxiety Rating Scale; MMSE, Mini-Mental State Examination; MoCA, Montreal Cognitive Assessment; UPDRS, Unified Parkinson's Disease Rating Scale.

* *This P-value indicates a statistically significant difference*.

#### Differences in Clinical Characteristics

All clinical data are shown in Table 2. The ET-PD group had an older mean age at onset of PD than the t-PD group (*P* = 0.004). Some non-motor features were significantly less common in the ET-PD group than the t-PD group, including constipation (*P* < 0.001), hyposmia (*P* = 0.044), and RBD (*P* = 0.033). The HAMD, HAMA, MMSE, and MoCA scores did not differ between the two groups. Concerning motor features, the proportion of patients with ET-PD with asymmetric motor symptoms was lower than that in the t-PD group (*P* < 0.001). Bradykinesia, rigidity, and postural abnormalities were less common in the ET-PD group than the t-PD group (both *P* < 0.001). Resting tremor was found in 73% of the ET-PD group, which was lower than that in the t-PD group (*P* = 0.024). Head tremor was more common in the ET-PD group than the t-PD group (*P* < 0.001). Mandibular tremor was less common in the ET-PD group than the t-PD group (*P* = 0.038).

#### Difference in Electrophysiological Results

The head and upper limbs' postural tremor amplitudes were significantly higher in the ET-PD group than those in the t-PD group (*P* = 0.022, 0.001, respectively; Table 2). To distinguish patients with ET-PD from t-PD, the cutoff value of head postural tremor amplitude was 477.50 μV, with a sensitivity of 57% and specificity of 90%. For the upper limbs, the postural tremor amplitude cutoff value was 393.00 μV, with a sensitivity of 86% and specificity of 60% (Table 3, Figure 1B).

**Table 3:** Sensitivity, specificity, and AUC of electrophysiological features in distinguishing ET-PD patients from ET and t-PD patients.

**Groups**	**Cutoff**	**Sensitivity**	**Specificity**	**AUC**	***P*-value**
**ET-PD vs. ET**					
Postural tremor frequency					
Head	5.20	67%	100%	0.775	0.023
Upper limbs	5.45	100%	83%	0.727	0.003
Lower limbs	5.98	100%	83%	0.701	0.160
**ET-PD vs. t-PD**					
Postural tremor amplitude					
Head	477.50	57%	90%	0.793	0.040
Upper limbs	393.00	86%	60%	0.716	0.004

*ET-PD, Parkinson's disease developed from essential tremor; t-PD, tremor-dominant Parkinson's disease; ET, essential tremor*.

## Discussion

ET-PD and ET/t-PD can be clinically difficult to differentiate because of overlapping motor and non-motor symptoms. This study showed that hyposmia and electrophysiological biomarkers (postural tremor frequencies and amplitudes) could distinguish patients with ET-PD from those with ET or t-PD.

Epidemiological and clinical evidence has supported the view that the lifetime risk of developing PD was higher in patients with ET than those without ET (23). A long latency could be up to 50 years (24). Like previous studies (6, 25), we found that the average latency for ET patients to develop PD was 12.30 ± 2.18 years. Furthermore, in the ET-PD group, the age of onset of ET tended to be older than that in the ET group, while the age of onset of PD was older than that in the t-PD group. There was no significant difference between the ET-PD and ET groups concerning sex, which is in accordance with a previous study (25) but conflicts with another study that reported a male predominance of ET-PD (26). It may need a further prospective study to explore the epidemiological characteristics of ET-PD.

In our cohort, the HAMD, HAMA, MMSE, and MoCA scores did not differ between ET-PD and ET/t-PD. However, constipation, hyposmia, and RBD were more common in the t-PD group than the ET-PD group, while hyposmia, rather than constipation or RBD, was more common in the ET-PD group than the ET group, which was consistent with previous studies (5, 25). Another study found no significant differences between patients with ET-PD and ET with regard to non-motor features (10). These conflicting results may be due to different mean durations of ET development into PD and/or the different methodologies used in each study. In our study, early non-motor features, especially the appearance of hyposmia, may indicate that ET is beginning to develop into ET-PD. Hyposmia may be an early symptom of ET-PD/PD and is associated with cellular damage in the olfactory bulb (27).

Among the motor features, we found that bradykinesia, rigidity, and postural abnormality were more common in patients with ET-PD than ET. Bradykinesia is the cardinal motor symptom in PD, which has also been reported in ET (4, 28). Several studies have shown that cerebellar dysfunction is involved in the pathophysiology of movement slowness in ET (4, 29). As in ET, the cerebellum is thought to be involved in the pathophysiology of bradykinesia in ET-PD, which is now considered a network disorder (30). Together with basal ganglia–cortical loops, the cerebellum may be involved in the execution of repetitive movements, which play a role in movement feedback and compensate for impaired basal ganglia function (30). Moreover, bradykinesia, rigidity, and postural abnormalities are all related to the parkinsonism, resulting from decreased dopaminergic transmission in the motor region of the striatum, involving connectivity of the globus pallidus to the cortico-basal ganglia-cerebello motor circuit (31). Several recent clinicopathological studies suggested that the dramatic loss of these dopaminergic neurons starts before the onset of motor symptoms (32). Maybe the appearance of motor features in patients with ET-PD is also related to the change in their dopamine levels and could be an early symptom of the conversion from ET to ET-PD.

EMG examination is a convenient and inexpensive tool to discriminate patients with ET-PD from ET/t-PD (33) compared with the magnetic resonance support vector machine (34). In accordance with previous studies (8, 26), resting tremor was significantly more common in the ET-PD group than the ET group in our study. Recent studies observed higher connectivity of the globus pallidus pars interna (GPi) and putamen to the cerebello–thalamic circuit (35) and an impairment of the basal ganglia–thalamocortical loop (36, 37). Another study showed that the globus pallidus, caudate nucleus, and supplementary motor area were specifically damaged in ET patients with resting tremor (38). These works suggested that dopaminergic loss in the pallidum might induce hyperactivity in the cerebello–thalamic circuit, leading to resting tremor. Since it was found that a subset of patients with ET eventually developed PD (5), we hypothesized that resting tremor may be a prominent feature of early-stage ET-PD, which may involve similar pathological loops as those in t-PD patients (10).

Besides resting tremor, we also observed that postural tremor frequencies of the head, upper limbs, and lower limbs were significantly lower in patients with ET-PD than those with ET. Furthermore, the head and upper limbs' postural tremor amplitudes were significantly higher in patients with ET-PD than those with t-PD. The cutoff values to distinguish patients with ET-PD from those with ET/t-PD have high sensitivity and specificity. Indeed, the exact central oscillators in the genesis of postural tremor in ET-PD are not fully understood. Some studies have concluded that postural tremor is triggered by the basal ganglia (39, 40) and mediated by the cerebello–thalamic–cortical network (41, 42). Tremor amplitude and frequency are primary quantitative indexes for diagnosing and monitoring of tremors. There is evidence that the tremor frequency decreases with time, which could be an essential factor leading to a deterioration of ET (43). Another study showed that patient's conditions directly affect neural oscillations related to tremor frequencies (44). Central oscillators control tremor frequency while peripheral nerves and muscles exert a modulatory influence on tremor amplitude. The reduction of tremor amplitude is accompanied by increased variability of tremor frequency due to the desynchronization of central oscillators (39). We observed that the postural tremor amplitudes were higher in the ET-PD group than those in the t-PD group, and that the postural tremor frequencies were lower in the ET-PD group than those in the ET group. We try to explain this phenomenon as a consequence of increasing the number of active central oscillators and an increased synchronization of central oscillators in the ET-PD group.

To our knowledge, very few studies were conducted to explore the quantitative electrophysiological biomarkers for ET-PD at present. Furthermore, this is also the highlight of our research. This study may be the first research about the clinical and electrophysiological characteristics of ET-PD and ET/t-PD in Chinese populations. However, our research also has some limitations. For example, the sample size of this study is small, and the definite diagnosis of these patients was not confirmed by the pathological results. Nonetheless, all patients were carefully evaluated by professional movement specialists during hospitalization.

## Conclusion

In the current study, we present the clinical characteristics to distinguish patients with ET-PD from those with ET, including the early appearance of hyposmia and motor symptoms. Our findings indicate that quantitative electrophysiological biomarkers, including a distinct frequency and amplitude of postural tremor, could be useful for the earlier recognition of ET-PD and beneficial to further patient treatment.

## Data Availability Statement

The raw data supporting the conclusions of this article will be made available by the authors, without undue reservation.

## Ethics Statement

The studies involving human participants were reviewed and approved by the Ethics Committee of Beijing Tiantan Hospital. The patients/participants provided their written informed consent to participate in this study.

## Author Contributions

All authors listed have made a substantial, direct and intellectual contribution to the work, and approved it for publication.

## Conflict of Interest

The authors declare that the research was conducted in the absence of any commercial or financial relationships that could be construed as a potential conflict of interest.
